# The Arf-GDP-regulated recruitment of GBF1 to Golgi membranes requires domains HDS1 and HDS2 and a Golgi-localized protein receptor

**DOI:** 10.1242/jcs.208199

**Published:** 2018-04-19

**Authors:** Douglas Quilty, Calvin J. Chan, Katherine Yurkiw, Alexandra Bain, Ghazal Babolmorad, Paul Melançon

**Affiliations:** Department of Cell Biology, University of Alberta, Edmonton, AB, Canada T6G 2H7

**Keywords:** Golgi, Arf, GTPase, GBF1, Membrane trafficking, Regulation

## Abstract

We previously proposed a novel mechanism by which the enzyme Golgi-specific Brefeldin A resistance factor 1 (GBF1) is recruited to the membranes of the *cis*-Golgi, based on *in vivo* experiments. Here, we extended our *in vivo* analysis on the production of regulatory Arf-GDP and observed that ArfGAP2 and ArfGAP3 do not play a role in GBF1 recruitment. We confirm that Arf-GDP localization is critical, as a TGN-localized Arf-GDP mutant protein fails to promote GBF1 recruitment. We also reported the establishment of an *in vitro* GBF1 recruitment assay that supports the regulation of GBF1 recruitment by Arf-GDP. This *in vitro* assay yielded further evidence for the requirement of a Golgi-localized protein because heat denaturation or protease treatment of Golgi membranes abrogated GBF1 recruitment. Finally, combined *in vivo* and *in vitro* measurements indicated that the recruitment to Golgi membranes via a putative receptor requires only the HDS1 and HDS2 domains in the C-terminal half of GBF1.

## Abstract (precis)

**Summary:** *In vivo* and *in vitro* experiments demonstrate Arf-GDP regulation of GBF1 recruitment to a heat-labile and protease-sensitive site on Golgi membranes. This recruitment requires the HDS1 and HDS2 domains.

## INTRODUCTION

The secretory pathway is required for the correct modification and targeting of secretory cargoes. This pathway is composed of a series of membrane-bound compartments with varying characteristics and functions, including the endoplasmic reticulum (ER), the ER-Golgi intermediate compartment (ERGIC), the Golgi complex, and the *trans*-Golgi network (TGN), among others (Bonifacino and Glick, 2004). The exact mechanism by which these distinct compartments are created and maintained remains unknown but it is generally assumed that the recruitment of protein factors is required to establish and define each compartment (Lippincott-Schwartz, 2011). Within the secretory pathway, the Golgi complex functions as a central organizing organelle (Emr et al., 2009; Farquhar and Palade, 1998). This central Golgi stack processes and facilitates the targeting of newly synthesized cargoes as they emerge from the ERGIC and traffic through to the TGN.

The ADP-ribosylation factor (Arf) family proteins regulate several aspects of secretory traffic (Donaldson and Jackson, 2011). Arfs are activated by guanine nucleotide exchange factors (GEFs) by facilitating the exchange of a bound GDP (inactive) for a GTP (active). Published work suggests that initial Arf association with membranes may depend on Arf receptors that are present in the Golgi membrane (Gommel et al., 2001; Honda et al., 2005). Activation locks Arfs in a membrane-bound conformation (Melançon et al., 2003), in which it has been shown to regulate various effectors that direct secretory traffic (Donaldson and Jackson, 2011).

Arf activation requires the activity of a diverse family of ArfGEFs (Casanova, 2007; Cox et al., 2004; Donaldson and Jackson, 2011; Nawrotek et al., 2016). The Sec7 family is unified by the presence of a central Sec7 catalytic domain (Sec7d) that facilitates the nucleotide exchange on Arf. Comparative genomic analysis has defined five mammalian subfamilies of ArfGEF that vary widely in size and domain composition (Cox et al., 2004; Mouratou et al., 2005). They include the small- and medium-sized cytohesins, EFA6s and BRAGs, as well as the larger GBF1 and Brefeldin A (BFA)-inhibited GEFs (BIGs) (Casanova, 2007; Jackson and Bouvet, 2014). Interestingly, the small- and medium-sized ArfGEFs additionally contain classic domains such as pleckstrin-homology and coiled-coil domains that facilitate membrane and protein interactions. In contrast, the large ArfGEFs, such as GBF1 and BIGs, do not contain these domains and, therefore, must be recruited to membranes by a different mechanism. Several reports implicate small GTPases in the recruitment of BIGs to the TGN (Galindo et al., 2016; McDonold and Fromme, 2014; Richardson and Fromme, 2012). Less is known about GBF1; however, GBF1 contains five highly conserved protein domains in addition to the Sec7d, one or more of which could be involved in Golgi recruitment.

Arf proteins are dependent on GTPase-activating proteins, or GAPs, for the stimulation of GTP hydrolysis and conversion to an inactive GDP-bound form (Donaldson and Jackson, 2011; East and Kahn, 2011; Wright et al., 2014). As such, the relative localization and the activities of GEFs and GAPs will, therefore, determine local levels of Arf-GDP. It is currently widely accepted that there are three Golgi-localized ArfGAPs, i.e. ArfGAP1, ArfGAP2 and ArfGAP3. ArfGAP2 and ArfGAP3 (ArfGAPs 2/3) are largely dissimilar from ArfGAP1 except for the presence of an N-terminal ArfGAP domain (Weimer et al., 2008). Moreover, ArfGAPs 2/3 are expected to have a specialized role in the formation of coatomer protein complex-coated I (COPI) vesicles (Weimer et al., 2008), which may preclude these ArfGAPs from producing pools of Arf-GDP capable of positively regulating GBF1 recruitment. Studies on the localization of the Golgi-localized ArfGAPs, specifically ArfGAP3, have yielded conflicting interpretations. Whereas ArfGAP1 appears to reside predominantly on the *cis*-Golgi membrane and ArfGAP2 seems to co-localize with both *cis*-Golgi and TGN markers (Pevzner et al., 2012), ArfGAP3 localization remains contentious. Some researchers have reported ArfGAP3 to be on *cis*-Golgi membranes (Frigerio et al., 2007; Weimer et al., 2008), whereas Randazzo and colleagues found ArfGAP3 exclusively at the TGN (Shiba et al., 2013).

In mammalian cells, Arf activation at Golgi and ERGIC membranes involves the Golgi-localized but largely cytosolic GBF1 (Manolea et al., 2008). GBF1 recruitment to Golgi membranes is required for Golgi maintenance and function and, as such, must be regulated. Previous *in vivo* imaging experiments have identified a novel Arf-GDP-stimulated mechanism for GBF1 recruitment to ERGIC and Golgi membranes (Quilty et al., 2014). This mechanism allows GBF1 to respond to increasing or decreasing levels of Arf-GDP in order to maintain a homeostatic level of Arf-GTP at the Golgi. Here, we extended those studies and developed a cell-free assay that established a requirement for a heat-labile and protease-sensitive site that is needed for the recruitment of GBF1 to Golgi membranes. We propose that this ‘receptor’ is crucial to establishing the identity of the Golgi and that of the ERGIC.

## RESULTS

### GBF1 recruitment is linked to Arf-GDP produced by ArfGAP1

**Fig. 1.**
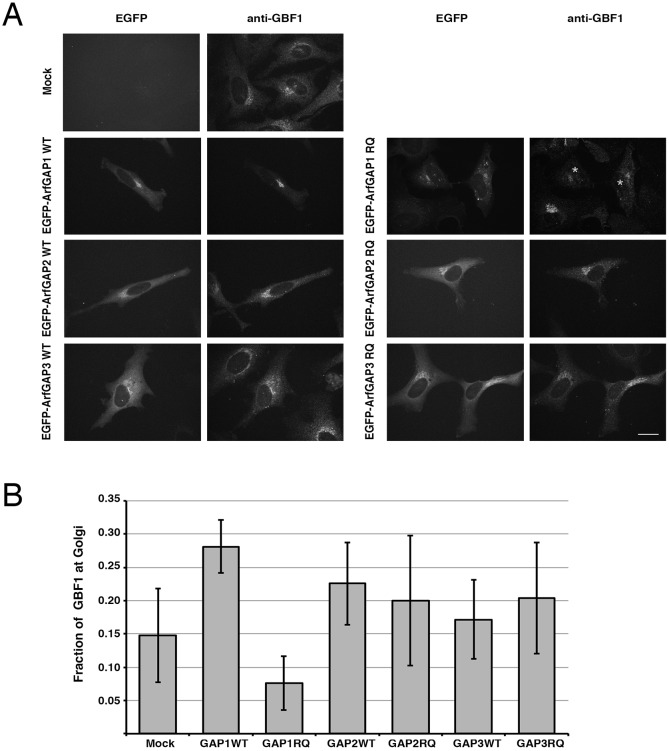
**ArfGAP1 expression alters GBF1 recruitment to Golgi membranes.** (A) HeLa cells were mock transfected or transfected with plasmids encoding EGFP-tagged forms of ArfGAP1 WT (GAP1WT), ArfGAP1 RQ (GAP1RQ), ArfGAP2 WT (GAP2WT), ArfGAP2 RQ (GAP2RQ), ArfGAP3 WT (GAP3WT) or ArfGAP3 RQ (GAP3RQ). Eighteen hours post transfection, cells were fixed and stained with mouse anti-GBF1 antibody and imaged using spinning-disc confocal microscopy. Expression levels of all EGFP-tagged proteins appeared similar as judged by signal intensity. Representative images are shown as projections of all *z*-slices. (B) Quantification was performed as described in Materials and Methods using a minimum of ten cells from each condition from each of the three independent experiments. The graph reports the fraction of endogenous GBF1 localized to the Golgi in cells that were mock-transfected or transfected with the indicated ArfGAPs. Error bars are ±s.d. (*n*=3). * indicate nuclei. Values were (0.09, 0.13, 0.23) for mock; (0.28, 0.24, 0.322) for GAP1WT; (0.070, 0.039, 0.118) for GAP1RQ; (0.19, 0.19, 0.30) for GAP2WT; (0.13, 0.16, 0.31) for GAP2RQ; (0.15, 0.12, 0.24) for GAP3WT; (0.14, 0.17, 0.30) for GAP3RQ. Scale bar: 26 μm.

Previously published *in vivo* experiments revealed that the overexpression of wild-type (WT) ArfGAP1 or its catalytically inactive R50Q (ArfGAP1 RQ) alters the amount of GBF1 bound to Golgi membranes (Quilty et al., 2014). Specifically, overexpression of WT ArfGAP1 results in increased GBF1 recruitment to Golgi membranes, whereas overexpression of the catalytically inactive mutant of ArfGAP1 causes a significant decrease in GBF1 at the Golgi. Here, we examined in more detail the ability of ArfGAPs to modulate GBF1 recruitment. We first tested whether ArfGAP1 altered GBF1 recruitment, preferentially relative to the Golgi-localized ArfGAP2 and ArfGAP3 (Weimer et al., 2008). To determine whether ArfGAP2 and/or ArfGAP3 play a role in the production of regulatory Arf-GDP, we transfected HeLa cells with WT or RQ mutant forms of ArfGAP1, ArfGAP2 and ArfGAP3. As previously observed (Quilty et al., 2014), expression of ArfGAP1 WT caused a clear increase in endogenous GBF1 levels on Golgi membranes, whereas ArfGAP1 RQ mutant expression had the opposite effect and resulted in a striking loss of GBF1 signal on Golgi membranes (Fig. 1A). The representative fields selected contained untransfected cells to better illustrate the dramatic impact of ArfGAP1 expression. To ascertain the reproducibility and significance of these observations, we quantified our imaging results by calculating the percent of endogenous GBF1 signal found within the Golgi area for 10 cells from three separate replicate experiments (30 cells in total for each condition) (Fig. 1B). This approach yields a more accurate quantification than the simpler Golgi:cytoplasm ratio previously reported by Quilty et al., 2014. This analysis demonstrated that overexpression of WT ArfGAP1 conferred ∼2-fold increase in Golgi-localized GBF1 staining, whereas expression of ArfGAP1 RQ resulted in a 50% reduction in Golgi-localized GBF1 staining, relative to mock-transfected cells. The ArfGAP1 WT induced a significant increase in GBF1 recruitment (*n*=3) relative to mock-treated cells. The most striking difference in GBF1 localization was observed between cells expressing ArfGAP1 WT and those expressing ArfGAP1 RQ (*n*=3).

In contrast, expression of ArfGAP2 WT and ArfGAP3 WT did not show a significant increase in GBF1 staining at the Golgi (Fig. 1A). More importantly, ArfGAP2 RQ and ArfGAP3 RQ expression did not decrease GBF1 staining at the Golgi. Quantification of the fraction of GBF1 on Golgi structures revealed that there was only a slight change in GBF1 localization when either WT or RQ mutant ArfGAP2 and ArfGAP3 proteins were expressed (Fig. 1B). Failure of ArfGAP2 and ArfGAP3 overexpression to impact on GBF1 levels at the Golgi suggests that they do not produce sufficient regulatory Arf-GDP. This could be due to either a lack of proximity to Arf-GDP-regulated elements or a specialized function of ArfGAP2 and ArfGAP3 that precludes their production of Arf-GDP capable of regulating GBF1 recruitment.

### TGN-restricted Arf-GDP does not alter GBF1 recruitment

Previous studies indicate that regulatory Arf-GDP must be membrane-associated since N-terminal myristoylation is essential to promote GBF1 recruitment (Quilty et al., 2014). In order to test the impact of Arf-GDP localization within the Golgi on GBF1 recruitment, we constructed a tagged form of an Arf1-Arf6-Arf1 chimera (Arf1-6-1) that has been shown to localize predominantly to the TGN (Honda et al., 2005). We first confirmed that the WT Arf1-6-1-GFP chimera, which contains amino acid residues 101-116 of Arf6, displayed TGN-restricted localization by analyzing transient expression in HeLa cells (Fig. S1). As predicted, the Arf1-6-1 chimera clearly co-localized with the TGN marker TGN46 and remained well resolved from the *cis*-Golgi marker p115. Line-scan analysis confirmed that Arf1-6-1-GFP preferentially localized to the TGN (Fig. S1B).

**Fig. 2.**
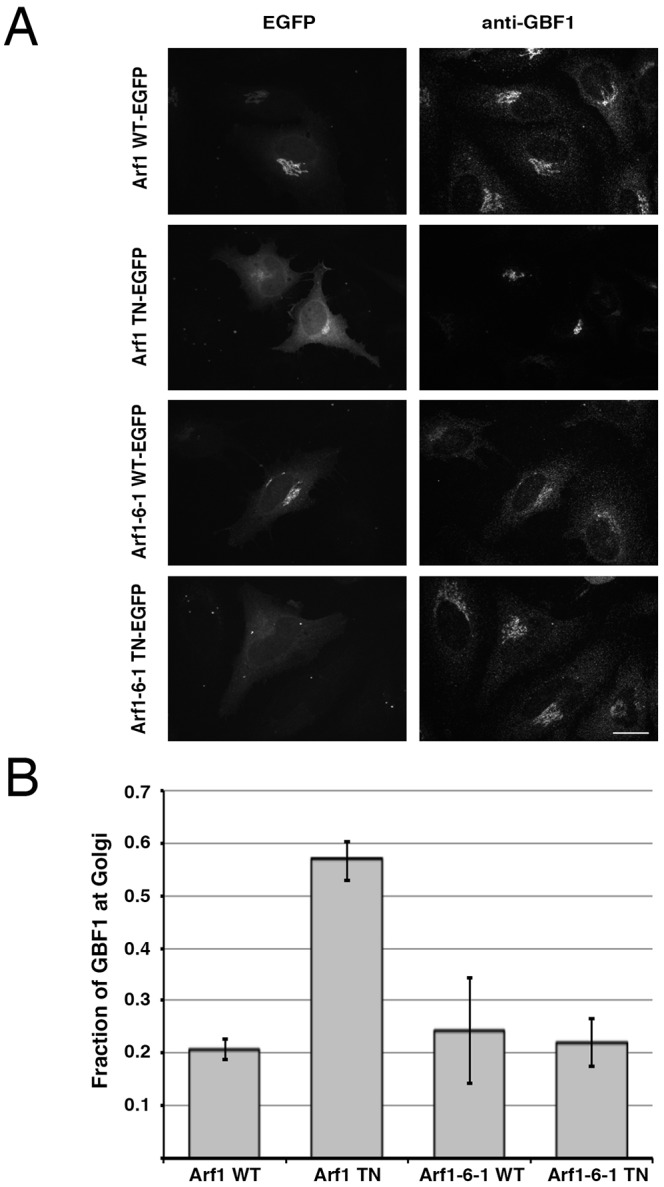
**Expression of TGN-localized Arf1-6-1 has no effect on GBF1 recruitment to Golgi membranes.** (A) HeLa cells were transfected with EGFP-tagged WT or mutant Arf1 or Arf1-6-1. Cells were fixed and stained with mouse anti-GBF1 antibody and images were collected using spinning-disc confocal microscopy. Representative images are shown as projections of all *z*-slices. (B) Quantification was carried out by selecting a minimum of 10 cells from each condition from each of the three separate experiments. The graph reports the fraction of GBF1 localized to the Golgi in cells that were transfected with WT or mutant Arf1 or Arf1-6-1 constructs. Error bars are ±s.d. (*n*=3). Values were (0.19, 0.28, 0.20) for Arf1WT; (0.59, 0.52, 0.58) for Arf1TN; (0.34, 0.25, 0.14) for Arf1-6-1WT; (0.22, 0.26, 0.17) for Arf1-6-1TN. Scale bar: 26 μm.

To test the ability of this Arf1-6-1 chimera to regulate GBF1 membrane association, we constructed and assessed the impact of expressing a GDP-arrested T31N mutant form (Arf1-6-1TN). HeLa cells were transfected with plasmids encoding the WT and T31N mutant form of either Arf1-6-1-GFP or Arf1-GFP. Cells were then fixed and stained to detect endogenous GBF1 (Fig. 2A). Imaging results of cells expressing low levels of the mutant Arf confirmed that expression of the T31N mutant of Arf1 causes a striking increase in GBF1 recruitment to Golgi membranes relative to the Arf1 WT control. In sharp contrast, expression of the Arf1-6-1 T31N construct failed to induce GBF1 recruitment. Quantification of the fraction of total GBF1 found on Golgi membranes confirmed that there was no significant increase in Golgi-localized GBF1 for the Arf1-6-1 T31N-expressing cells relative to Arf1-6-1 WT-transfected controls (Fig. 2B). As predicted, cells expressing Arf1 T31N displayed a 2.5-fold increase in Golgi-localized GBF1 relative to those expressing Arf1 WT (*P*<0.0005, *n*=3). These data suggest that association of regulatory Arf-GDP with *cis*-Golgi membranes is required to promote GBF1 recruitment to Golgi membranes.

### GBF1 recruitment to Golgi membranes *in vitro* is temperature sensitive

To provide further evidence for a role for Arf-GDP in the regulation of GBF1 recruitment to Golgi membranes, we performed *in vitro* GBF1 recruitment experiments. To establish an *in vitro* GBF1 recruitment assay, we utilized a preparation method for highly stacked Golgi-enriched membranes (WNG) from rat liver (Dominguez et al., 1999) and that contained significant levels of bound GBF1 (Gilchrist et al., 2006). We first confirmed by centrifugation and anti-GBF1 immunoblotting that WNG membranes contained bound GBF1 (Fig. S2). This analysis established that WNG membranes contained a readily detected band at the expected size of 250 kDa, almost exclusively associated with the pellet under our assay conditions. These data suggest that the WNG fraction constitutes a viable source of membranes for an *in vitro* GBF1 recruitment assay. We used cytosol produced from the well-studied normal rat kidney (NRK) cell line expressing GFP-GBF1 (Zhao et al., 2006) as a source of GBF1 for the assay since we were unable to produce full-length recombinant GBF1. Lastly, binding assays were carried out in the presence of excess protease inhibitors as both endogenous and exogenous GBF1 proved extremely sensitive to proteolysis.

**Fig. 3.**
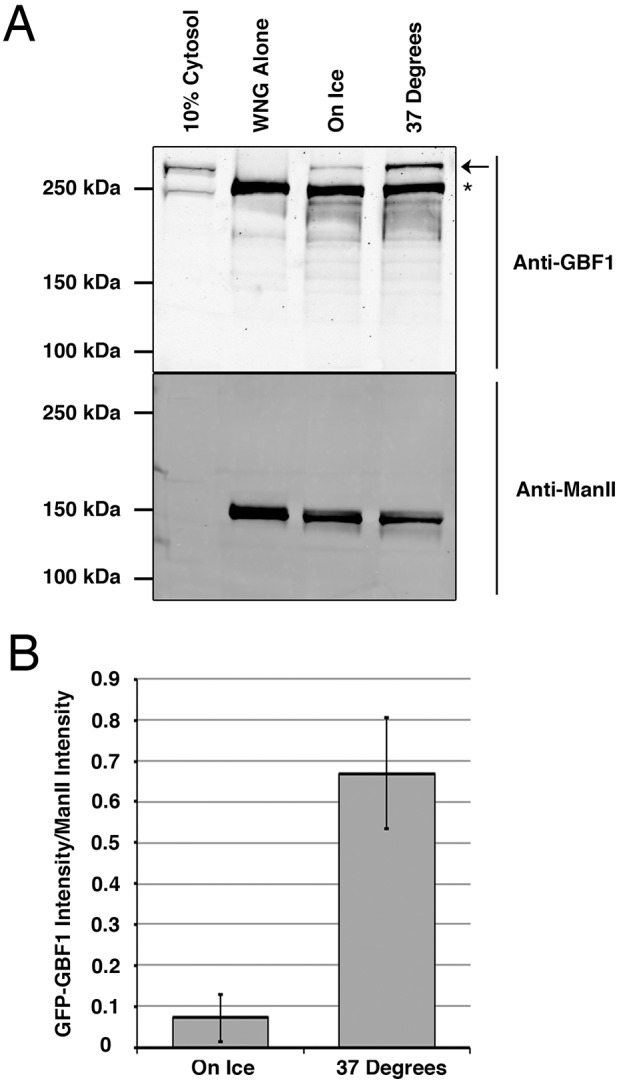
**Reconstitution of GBF1 recruitment to Golgi membranes in a cell-free assay.** (A) WNG membranes recruit GBF1 at physiological temperature, but not on ice. WNG membranes were incubated with GFP-GBF1 NRK cell cytosol at 37°C or on ice for 5 min and then separated into membrane and supernatant fractions by centrifugation. Resulting pellets were separated by SDS-PAGE along with 10% cytosol and WNG controls (WNG Alone). Proteins were transferred to nitrocellulose and incubated with a mouse anti-GBF1 monoclonal antibody and rabbit anti-ManII antibodies, then incubated in donkey anti-mouse Alexa Fluor 750 and donkey anti-rabbit Alexa Fluor 680 secondary antibodies. The resulting immunoblot was then scanned in a Licor Odyssey scanner. A representative blot is displayed. The arrow and the asterisk mark the positions of GFP-tagged and endogenous GBF1, respectively. (B) GFP-GBF1 band intensities were quantified and corrected for the amount of WNG present by normalization to the ManII band intensity as described in Materials and Methods. Error bars are ±s.d. (*n*=3). Values 0.10, 0.11 and 0.01 were obtained for WNG membranes on ice, and 0.83, 0.59 and 0.59 were obtained for those at 37°.

To measure recruitment of GFP-GBF1 from the cytosol onto the WNG membranes, we incubated cytosol of NRK cells expressing GFP-GBF1 with the membranes for 5 min either on ice or at 37°C, as described in Materials and Methods. Following incubation, samples were separated by centrifugation and analyzed by immunoblotting, as described in Materials and Methods (Fig. 3). The resulting immunoblots (Fig. 3A) clearly demonstrate that GFP-GBF1 (arrow) was recruited to WNG membranes and that greater levels of recruitment occurred when assays were performed at 37°C, rather than on ice.

Quantification of band intensity using Licor Odyssey software allowed for the corrected measurement of GFP-GBF1 recruitment in three replicate experiments. Results were normalized to the Golgi protein Mannosidase II (ManII) to correct for the amount of membrane recovered in each assay. ManII levels in these samples were not significantly different. The ratio of GFP-GBF1 band intensity to ManII band intensity was calculated for each replicate, and values are given as the means±s.d. (*n*=3) (Fig. 3B). The quantification indicated a striking 6.5-fold increase in GFP-GBF1 recruited at 37°C relative to that recruited at 0°C. These results strongly suggest that GBF1 recruitment to membranes involves a temperature-sensitive step.

### Arf-GDP stimulates GBF1 recruitment to Golgi membranes *in vitro*

**Fig. 4.**
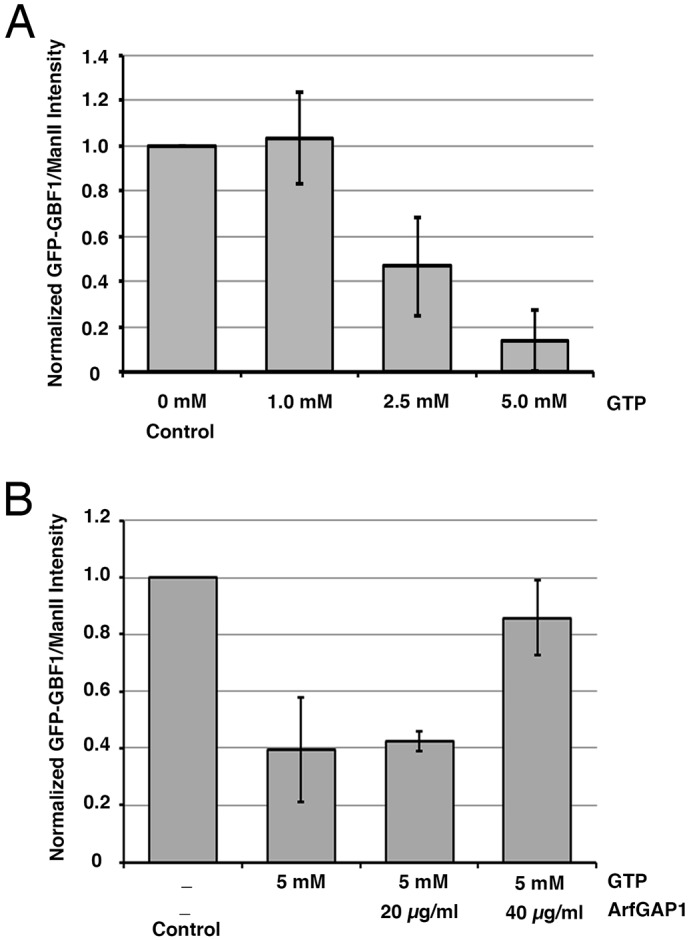
***In vitro* recruitment of GBF1 can be modulated by addition of GTP and ArfGAP1.** (A,B) WNG membranes were incubated with GFP-GBF1 NRK cell cytosol at 37°C, with or without the addition of GTP or ArfGAP1, as indicated. Samples were then separated into membrane and supernatant fractions by centrifugation. The resulting pellets were separated by SDS-PAGE with 10% cytosol and WNG membranes, and analyzed as described in Materials and Methods. The resulting immunoblots were then scanned using a Licor Odyssey scanner, and GFP-GBF1 band intensity was quantified as described in Materials and Methods. See Fig. S3 for a representative blot. Values obtained were then normalized to control values to determine the fold-change relative to control. The resulting quantification is displayed; error bars are ±s.d. (*n*=3). Values for panel A were (1, 1, 1) for control; (1.21, 1.08, 0.81) for 1 mM GTP; (0.70, 0.43, 0.27) for 2.5 mM GTP; and (0.29, 0.03, 0.1) for 5 mM GTP. Values for panel B were (1 ,1, 1) for control; (0.22, 0.58, 0.39) for GTP; (0.43, 0.46, 0.39) for GTP/20 µg/ml ArfGAP1; and (0.72, 0.87, 0.98) for GTP/40 µg/ml ArfGAP1.

Previous *in vivo* work predicted that recruitment of GBF1 to WNG membranes *in vitro* should respond to changes in Arf-GDP levels (Quilty et al., 2014; Fig. 1). We tested this prediction in *in vitro* GBF1 recruitment assays by adding an excess amount of GTP, which should stimulate conversion of Arf-GDP to Arf-GTP. Binding assays revealed that much less GFP-GBF1 was recovered on Golgi membranes in the presence of excess GTP (Fig. 4A; see Fig. S3A for a representative immunoblot). The ratio of GFP-GBF1 to ManII band intensity was calculated and normalized for each replicate to control. This quantification indicated that there was a progressive and significant reduction, to ∼10%, in the amount of GFP-GBF1 recruited to Golgi membranes in the presence of 5 mM GTP relative to control samples (Fig. 4A). These data indicate that GBF1 recruitment to Golgi membranes *in vitro* is sensitive to guanine nucleotide, likely through Arf activation and the consequent reduction in Arf-GDP.

To confirm that the reduction in GBF1 recruitment in the presence of excess GTP results from Arf activation, we examined whether addition of recombinant ArfGAP1 can reverse the effect of GTP. *In vitro* GBF1 recruitment assays were performed in the presence of 5 mM GTP alone, or with either 20 or 40 μg/ml recombinant ArfGAP1 (Fig. 4B; see Fig. S3B for a representative immunoblot). The ratio of GFP-GBF1 to ManII band intensity was calculated for each replicate and normalized to the control. This quantification indicated that addition of 40 μg/ml recombinant ArfGAP1 conferred ∼50% recovery in GFP-GBF1 recruitment relative to control and 5 mM GTP conditions. These data suggest that increased Arf-GDP levels positively regulate *in vitro* recruitment of GBF1 to Golgi membranes.

### Recruitment of GBF1 to WNG membranes involves a heat-labile and protease-sensitive ‘receptor’

**Fig. 5.**
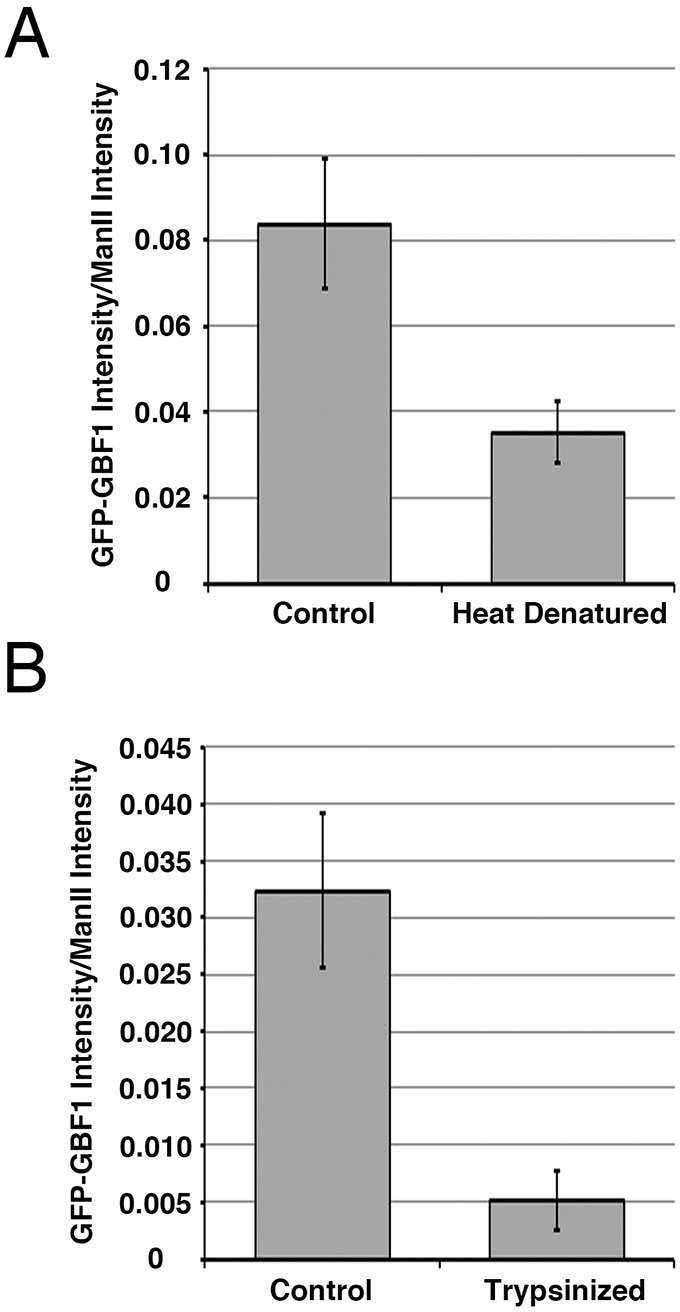
**Recruitment of GBF1 to WNG membranes involves a heat-labile and protease-sensitive ‘receptor’.** (A) Heat denaturation of WNG abrogates GBF1 recruitment to WNG membranes. WNG membranes were pre-incubated at 95°C or on ice, then subjected to the binding assay as for Figs 4 and 5, and then separated into membrane and supernatant fractions by centrifugation. The resulting pellets were separated by SDS-PAGE along with 10% cytosol and WNG membranes and analyzed as described in Materials and Methods. The resulting immunoblots were then scanned in a Licor Odyssey scanner and the GFP-GBF1 band intensity was quantified as described in Materials and Methods. See Figs S3 and S4 for representative blots. The resulting quantification is displayed; error bars are ±s.d. Values were (0.091, 0.066, 0.093) for control and (0.027, 0.037, 0.041) for heat-denatured membranes. (B) Pre-treatment of WNG with trypsin greatly reduces GBF1 recruitment to WNG. WNG membranes were incubated with GFP-GBF1 NRK cell cytosol at 37°C following incubation with or without trypsin and then separated into membrane and supernatant fractions by centrifugation. The resulting pellets and supernatants were separated by SDS-PAGE along with 10% cytosol and WNG-alone controls and analyzed as described in Materials and Methods (see Fig. S4 for representative blots). Analysis of supernatant fractions confirmed that 70% of GFP-GBF1 remained in trypsin-treated samples (see Fig. S4). The GFP-GBF1 band intensities were quantified and normalized to the ManII band intensity. The resulting quantification is displayed; error bars are ±s.d. Values were (0.035, 0.025, 0.037) for control and (0.003, 0.005, 0.008) for trypsin-treated membranes.

The nature of the Golgi ‘receptor’ onto which GBF1 is recruited remains unknown – it could be a lipid, soluble protein, transmembrane protein or a combination of the above. To test the possibility that recruitment involves a protein, we first treated WNG membranes at elevated temperature prior to testing for GBF1 recruitment. WNG membranes were incubated either on ice or at 95°C for 5 min and then assayed for *in vitro* GBF1 recruitment as before (see Fig. S4A for a representative immunoblot**)**. Quantification of three separate experiments (Fig. 5A) revealed a >50% reduction in GFP-GBF1 recruitment to heat-denatured WNG relative to control membranes (*P*<0.01, *n*=3).

To directly test the presence of a Golgi-localized GBF1 protein receptor, we treated WNG membranes with trypsin prior to performing the recruitment assay. We incubated WNG membranes for 5 min at 37°C either with or without 0.5 mg/ml trypsin. Following incubation, 1.0 mg/ml soybean trypsin inhibitor was added to control and trypsin-treated WNG membranes, which were then incubated on ice. Resulting membranes were then used for *in vitro* GBF1 recruitment assay, as previously described. Quantification of three replicate experiments was performed and the GFP-GBF1 band intensity was normalized to the ManII band intensity (*n*=3), as before (Fig. 5B; see Fig. S4B for a representative immunoblot). Samples containing membranes pre-treated with trypsin displayed a near-complete loss in GFP-GBF1 recruitment. Quantification of three separate replicates indicated a striking 85% reduction in GFP-GBF1 recruitment in trypsin-treated WNG samples relative to control WNG (*n*=3). Analysis of supernatant fractions confirmed that GFP-GBF1 remained available in the cytosol for recruitment (Fig. S4B). The striking reduction in GFP-GBF1 recruitment observed cannot be completely attributed to degradation, and we conclude that trypsin treatment of WNG abrogates GBF1 recruitment. The data from Fig. 5 clearly indicate that Golgi-localized protein(s) are required for recruitment of GBF1 to Golgi membranes. It remains unclear, however, whether these unknown protein(s) function as direct binding partner or, perhaps, as enzyme required for modification(s) that promote GBF1 recruitment.

### Identification of a minimal Golgi-binding domain in the C-terminal half of GBF1

**Fig. 6.**
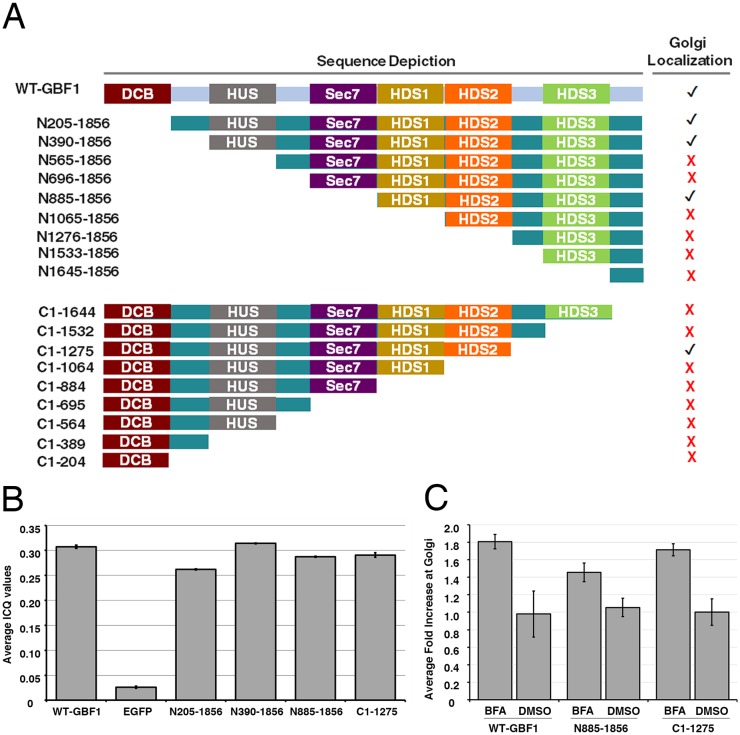
**Generation and analysis of GBF1 truncations.** (A) Schematic diagram of the pEGFP-GBF1 truncation library. EGFP-tagged constructs were generated by PCR and inserted into a modified pEGFP-SbfI vector as described in Materials and Methods. Constructs were N-terminally tagged with EGFP and named N- (N-terminal deletions) or C- (C-terminal deletions) followed by the corresponding amino acid range present in each. On the right, Golgi localization (✔) is defined as a significant overlap with mCherry-ERGIC-53. See images in Fig. S6. (B) Intensity Correlation Quotient (ICQ) analysis confirms the localization of several truncations to the Golgi. Images similar to those shown in Fig. S6 were analyzed for intensity correlation between the GFP and mCherry signals using Fiji (version 2.0.0-rc-43/1.51 g; build 49b667f9aa). A minimum of eight cells from each of the three separate replicates were quantified. This analysis yielded mean ICQ. Mean ICQ values±s.d. are reported here for each construct that localized to the Golgi. Pairwise *t*-test comparison between ICQ values of GFP and the GBF1 chimeras yielded *P*<0.001. (C) BFA stimulates Golgi recruitment of several truncated GBF1 variants. HeLa cells were transfected with plasmids encoding either GFP, full-length GFP-GBF1 or the indicated truncations, and then imaged for 6 min by live-cell wide-field fluorescence microscopy. BFA was added to a concentration of 10 µg/ml 1 min after the start of imaging. Several representative fields at 90 s post-BFA addition were captured. The fraction of GBF1 on juxta-nuclear structures was quantified from 8-10 cells per replicate, as described in Materials and Methods. Mean values are displayed±s.d. (*n*=3). Values were (1.87, 1.65, 1.89) for WT GBF1 BFA; (1.27, 0.77, 1.20) for WT GBF1 DMSO; (1.47, 1.47, 1.43) for N885-1856 BFA; (1.07, 0.93, 1.13) for N885-1856 DMSO; (1.56, 1.74, 1.83) for C1-1275 BFA; (0.92, 1.16, 1.20) for C1-1275 DMSO. See representative images in Fig. S7.

To characterize the interaction of GBF1 with *cis*-Golgi membranes, we chose to first identify domains of GBF1 required for this process. We constructed a set of GFP-tagged proteins (the pEGFP-*Sbf*I-GBF1 truncation library), in which each of the six conserved domains and/or inter-domain regions of GBF1 were progressively deleted from either the N- or the C-terminal end (Fig. 6A). The library included nine N-terminal truncations and nine C-terminal truncations, based on the borders of the DCB, HUS, Sec7, HDS1, HDS2 and HDS3 domains initially described by Cherfils and colleagues (Mouratou et al., 2005).

To assess the ability of each truncation to be recruited to Golgi membranes, HeLa cells were co-transfected with plasmids encoding mCherry-ERGIC-53, and either members of the truncation library, full length GFP-GBF1 (WT) or GFP. Initial experiments with all truncations established that localization studies were best performed in live cells as fixation often yielded discrepant results (an example with truncation N885-1856 is provided in Fig. S5). Further experiments revealed inconsistent loss in Golgi localization of GBF1 truncations when performing analysis in cells treated with a variety of fixatives, from alcohols to high concentrations of paraformaldehyde. Cells expressing each truncation were then imaged by live cell epifluorescence microscopy, as described in Materials and Methods, to assess whether each truncation localized to a juxta-nuclear Golgi structure detected with mCherry-ERGIC-53. A summary of this localization analysis is provided (Fig. 6A). Image analysis clearly showed that, of the 18 truncations queried, only four displayed a clear juxta-nuclear localization (Fig. S6). Specifically, truncations N205-1856, N390-1856, N885-1856 and C1-1275 displayed localization to membranes positive for mCherry-ERGIC-53, suggestive of Golgi recruitment. Intensity correlation analysis, performed as described by Li et al. (2004), confirmed that, contrary to soluble GFP, all four truncations associated with Golgi structures exhibited intensity correlation quotients (ICQs) similar to those of full length GBF1 (Fig. 6B). Pairwise *t*-test analysis of >35 cells from three separate experiments between GFP and each GBF1-chimera confirmed the significance (*P*<0.001) of this result. Importantly, cells expressing non-Golgi-localized truncations displayed clearly labeled juxta-nuclear ERGIC-positive structures, suggesting that, despite their lack of Golgi localization, they did not negatively impact on Golgi maintenance. These observations further suggest that the regions around the Sec7 and HDS3 domains act negatively regarding the membrane localization of GBF1.

The observation that several truncation mutants containing the DCB and Sec7 domains (e.g. C1-884) failed to recruit to Golgi structures prompted us to determine whether BFA addition can drive Golgi association. Experiments similar to those described in Fig. S6 were repeated. In these experiments, images were captured both before and after a brief 90 s treatment with either carrier DMSO or 10 µg/ml BFA (Fig. S7). The results revealed that the presence of BFA promoted further recruitment of all Golgi-localized truncations. Importantly, BFA at 10 µg/ml had no detectable effect on any of the other 14 truncations. The fold-increase in GBF1 recruitment in the presence of BFA was quantified for the smallest member of the N- and C-terminal truncations that localized to the Golgi, as described in Materials and Methods (Fig. 6C). This analysis demonstrated that the presence of a Sec7 domain was neither essential for detection of Golgi association in the absence of BFA nor required to observe stimulation by the drug; it further suggested that regulatory Arf-GDP acts, directly or indirectly, on a domain other than Sec7d. Interestingly, the only domains common to the four Golgi-associated constructs were HDS1 and HDS2. None of the other domains appeared necessary for Golgi localization.

**Fig. 7.**
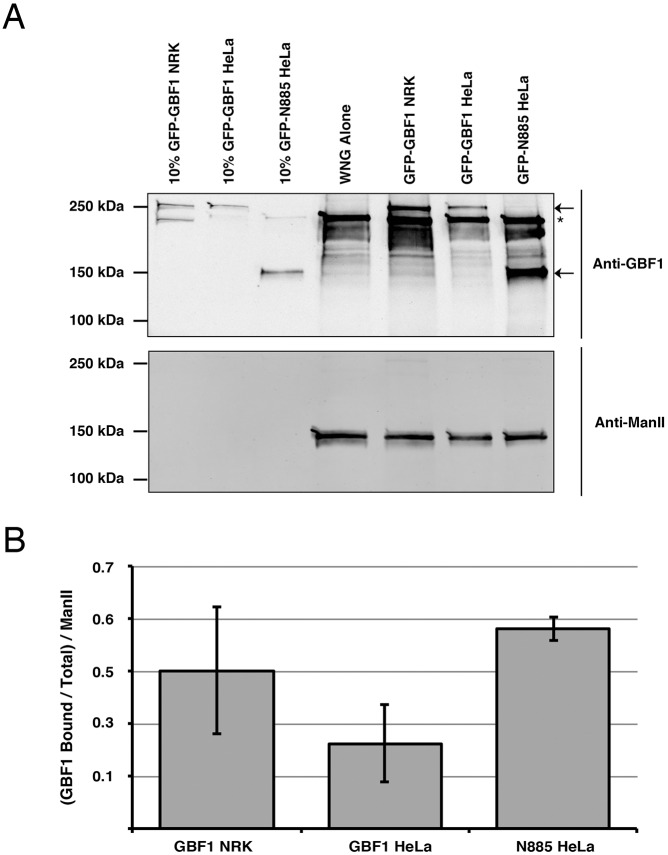
**GBF1 truncation N-885**-**1856** **lacking the N-terminus but containing domains HDS1 and HDS2 associates with Golgi membranes *in vitro*.** (A) WNG membranes recruit truncation N-885-1856 at physiological temperature. WNG membranes were incubated with cytosol obtained either from NRK cells or from HeLa cells stably expressing either full length GBF1 or truncation N-885-1856 and processed as for Fig. 3. The resulting pellets were run by SDS-Page along with 10% cytosol and WNG alone controls. A representative blot is displayed. The arrows and the asterisk mark the positions of GFP-tagged and endogenous GBF1, respectively. (B) GFP-GBF1 band intensities were quantified and corrected for the amount of WNG present by normalization to the ManII band intensity as described in Materials and Methods. The resulting quantification is displayed with standard error of the mean (*n*=3). Values were (0.82, 1.84, 1.87) for GBF1 NRK; (0.43, 1.17, 0.84) for GBF1 HeLa; and (2.02, 1.80, 1.90) for N885 HeLa.

To confirm the *in vivo* results, we chose to perform *in vitro* membrane-binding assays. Binding experiments were performed as those shown in Fig. 3 with the exception that the cytosol was prepared from HeLa cells stably expressing either full length GFP-GBF1 or its smallest truncated form (N885-1856). The results demonstrate binding to Golgi membranes of the HDS1- and HDS2-containing C-terminal fragments (Fig. 7**)**. Such binding was reproducible, as similar results (±5%) were obtained in several replicates. In combination, the *in vivo* and *in vitro* results strongly suggest that domains HDS1 and HDS2 are both necessary for interaction with Golgi membranes.

## DISCUSSION

Our previous *in vivo* study had elucidated a substrate-stimulated, feed-forward mechanism in which Arf-GDP promotes GBF1 recruitment to Golgi membranes that results in Arf-GTP homeostasis (Quilty et al., 2014). Here, we extended those observations both *in vivo* and *in vitro* to yield evidence for a heat-labile and protease-sensitive receptor on Golgi membranes. We first reported two sets of observations that suggest that the source of the Arf-GDP matters. ArfGAP1 was particularly effective at altering GBF1 recruitment relative to ArfGAP2 and ArGAP3. The T31N form of Arf1, but not the TGN-restricted Arf1-6-1, stimulated GBF1 recruitment. Cell-free reconstitution of GBF1 recruitment allowed us to confirm and extend these results. In vitro binding assays carried out in the presence of GTP and ArfGAP1 confirmed that recruitment of GBF1 is tightly linked to the level of Arf-GDP at the Golgi membrane. More importantly, the cell-free assay allowed us to examine the impact of various treatments on the recruitment reaction and to establish the presence of a heat-labile and protease-sensitive site on Golgi membranes that is required for efficient recruitment. Lastly, *in vivo* and *in vitro* experiments established that Arf-GDP-regulated membrane recruitment did not require the DCB, HUS or Sec7 domains but, instead, involved domains of GBF1 present in the C-terminal half of the protein.

### Recruitment of GBF1 to the Golgi requires HDS1 and HDS2 domains but not DCB, HUS and Sec7 domains

The demonstration that heat and protease treatments of Golgi-enriched fractions reduce GBF1 recruitment strongly suggests the presence of a membrane-associated receptor on *cis*-Golgi membranes. As a first step toward characterization of this interaction, we examined in live cells the recruitment of GBF1 truncations constructed exactly according to the boundaries defined by Cherfils and colleagues (Mouratou et al., 2005). Interestingly, truncations lacking the N-terminal half of GBF1, including the DCB, HUS and Sec7 domains, associated with the Golgi in a BFA-sensitive manner. Furthermore, none of the C-terminal truncations lacking HDS2 localized to the Golgi, even in the presence of BFA. Lastly, recruitment of the N885-1856 GBF1 to Golgi membranes was observed *in vitro*.

**Fig. 8.**
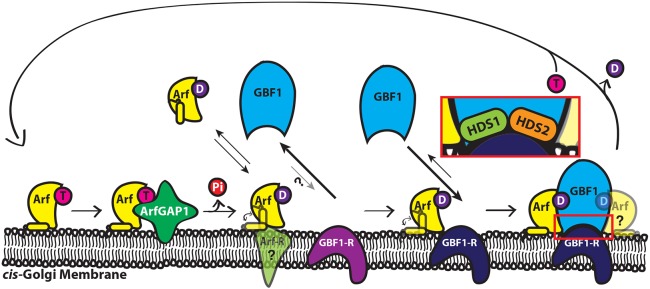
**‘Arf-increase’ model for the regulation of GBF1 recruitment to *cis*-Golgi membranes.** Arf-GDP acts as a trigger to recruit GBF1 to Golgi membranes, which involves GBF1 domains HDS1 and HDS2. Regulatory Arf-GDP can be produced through hydrolysis of Arf-GTP by ArfGAP1 or can be recruited directly from the cytosol. Arf-GDP can be either free or bound to an unknown receptor. GBF1 is recruited from the cytosol through domains HDS1/HDS2 to a no-affinity or low-affinity receptor (magenta) that likely requires Arf-GDP for activation (dark blue). The nature of the binding site for regulatory Arf-GDP remains unknown, although it must be present at *cis*-Golgi membranes, possibly at the GBF1 receptor itself. However, we cannot eliminate the possibility that Arf-GDP regulates a lipid-modifying enzyme to cause GBF1 recruitment. This self-limiting model provides a mechanism to maintain homeostatic levels of Arf-GTP. T, GTP; D, GDP; Pi, inorganic phosphate.

These results were surprising, as previous reports had suggested that the DCB and HUS domains are required for Golgi binding (Bouvet et al., 2013). After fixation, we observed a similar lack of Golgi localization of truncations without the DCB and HUS domains; however, our attempts to solve localization issues resulting from fixation proved unsuccessful. We speculate that the negative observations resulted from a fixation artefact. A recent report on DCB-mediated dimerization also suggests that the N-terminal DCB domain is not required for Golgi recruitment (Bhatt et al., 2016). Our own analysis of additional GBF1 truncations, both *in vivo* and *in vitro*, confirms the original conclusion of Jackson and colleagues, i.e. that HDS1 and HDS2 domains are crucial for GBF1 association with Golgi membranes (Bouvet et al., 2013). It is important to note that recruitment of the smallest truncation lacking the catalytic Sec7 domain remained BFA sensitive. We propose that domains HDS1 and HDS2 are both necessary to direct GBF1 recruitment and association with the Golgi (Fig. 8).

### Localization of regulatory Arf-GDP matters

We tested the impact of Arf-GDP localization by using two separate approaches. We first examined the impact of the GDP-arrested form of the Arf1-6-1 chimera previously shown to localize primarily to the TGN (Honda et al., 2005). This chimera contains primarily Arf1 sequences but the replacement of 16 residues (101-116) from Arf6 causes the protein to localize primarily on TGN membranes, well resolved from either the *cis*-Golgi or the plasma membrane. We constructed an Arf1 T31N mutant and found it to be largely soluble and not to alter GBF1 distribution. In sharp contrast, a similar mutation in Arf1 caused dramatic accumulation of GBF1 on juxta-nuclear Golgi membranes. The lack of impact reported in Fig. 2 was clear and significant. These results strongly suggest that restriction of the Arf1-6-1 chimera to the TGN prevents feedback on GBF1 recruitment. Note that we cannot exclude the possibility that Arf1 residues 101 to 116 of Arf1 are crucial to regulation.

The second approach compared the impact of overexpressing WT or catalytically dead forms of ArfGAP1, ArfGAP2 and ArfGAP3. We have previously reported that overexpression of the *cis*-Golgi-localized ArfGAP1 WT results in a significant change in the ratio of free to Golgi-bound GBF1 (Quilty et al., 2014). Here, we have extended these observations to all three ArfGAPs and used a more accurate quantification of the fraction of GBF1 signal at the Golgi. Whereas transfection of active ArfGAP1 caused a significant increase in GBF1 recruitment, transfection of the catalytically inactive R50Q mutant of ArfGAP1 led to a striking reduction in Golgi-localized GBF1. As ArfGAP1 overexpression increases Arf-GDP production (Cukierman et al., 1995), we conclude that stimulation of GBF1 recruitment in response to overexpression of ArfGAP1 is a result of increased hydrolysis of Arf-GTP to Arf-GDP. Interestingly, overexpression of WT, or that of ArfGAP2 RQ or ArfGAP3 RQ, did not cause a significant change in GBF1 recruitment. These observations suggest that the Arf-GDP produced by different ArfGAP species has different impacts on GBF1 recruitment. In the case of ArfGAP3, this observation likely results from its localization to the TGN (Shiba et al., 2013). However, we cannot exclude that our observation results from lower GAP activity. For example, ArfGAP1 may be more efficient at promoting hydrolysis of Arf-GTP to Arf-GDP, which can regulate GBF1 recruitment on *cis*-Golgi membranes.

In addition to the classic role of GBF1 in Arf activation at the ERGIC and Golgi, multiple alternative localizations have been reported. Specifically, GBF1 has been implicated in endocytosis of glycosylphosphatidylinositol-anchored protein (GPI-AP)-enriched early endosomal compartments (GEECs) at the plasma membrane and in lipid droplet homeostasis (Bouvet et al., 2013; Gupta et al., 2009). The mechanism that governs GBF1 recruitment and localization at these subcellular compartments remains unclear. However, under conditions where Arf-GDP levels are increased, we never observed increased localization of GBF1 to the plasma membrane, which suggests that any potential GBF1 localization to the plasma membrane is not regulated by altered Arf-GDP levels.

### Regulation of ArfGEFs by Arf-GDP and Arf-GTP

Whereas our results clearly demonstrate a regulation of GBF1 recruitment by substrate Arf-GDP, it appears that the activity of other ArfGEFs is regulated by the GTP-bound form of small G proteins. For example, studies performed on BIG1 and the related yeast homolog Sec7p concluded that these ArfGEFs are recruited to Golgi membranes through interactions with the GTP-bound form of both Arf1 and Arl1 (Galindo et al., 2016; Richardson et al., 2012). Interestingly, regarding regulation of Sec7p, Arf1-GTP alleviates an autoinhibitory conformation mediated by the HDS1 domain, like that observed for ARF nucleotide-binding site opener (ARNO) (Stalder et al., 2011). This is in sharp contrast to the Arf-GDP-stimulated recruitment of GBF1 to the ERGIC and *cis*-Golgi membranes.

It is interesting that Arf1-GTP, the product of enzymatic activity, recruits both Sec7p and ARNO. The mechanism proposed for Sec7p/BIGs and ARNO results in a run-away ArfGEF activity due to Arf1-GTP stimulating a massive feed-forward recruitment further stimulated by enzymatic activity. These predictions fit with the observation that BIGs display high affinity for TGN membranes and do not have a significant cytosolic pool (Manolea et al., 2008). In contrast, involvement of substrate Arf-GDP in GBF1 recruitment would allow maintenance of a Golgi stack at steady-state, whereas production of Arf-GTP could promote BIG recruitment at the TGN. In other words, stimulation of GBF1 recruitment by Arf-GDP would lead to Arf activation and, ultimately, stimulation of BIGs activity. Contrary to the hypothesis presented by Sztul and colleagues (Lowery et al., 2013), GBF1 activity does not need to be localized to the TGN. In this model, long-term treatment with the GBF1 inhibitor Golgicide A causes TGN fragmentation and dispersal, as reported (Saenz et al., 2009).

### Concluding remarks

The molecular mechanisms that mediate the ‘activation’ of the GBF1 ‘receptor’ at *cis-*Golgi membranes remain elusive. However, in combination with the previous demonstration that the soluble G2A Arf1 mutant fails to regulate GBF1 (Quilty et al., 2014), we conclude that Arf-GDP promotes GBF1 recruitment when associated with membranes of the *cis*-Golgi and ERGIC. Arf-GDP likely regulates a Golgi-localized, proteinaceous GBF1 ‘receptor’ to promote its recruitment. This regulation could be direct, by inducing a conformational change that increases in affinity for GBF1, or indirect, by promoting post-translational modification. Note that our results do not exclude the possibility that the putative GBF1 receptor integrates several signals from a variety of proteins and/or lipids (Wright et al., 2014) or that it is composed of multiple proteins, including both transmembrane and peripheral proteins. Indeed, the recruitment of GBF1, as proposed for other GEFs (Richardson et al., 2012), could respond simultaneously to several stimuli, such as the phosphoinositide level, Rab1 (Alvarez et al., 2003) and receptor abundance/modification. A better understanding of the mechanism(s) of GBF1 recruitment will require identification of interaction partners.

## MATERIALS AND METHODS

### Cell culture and reagents

BFA was purchased from Sigma-Aldrich (St Louis, MO) and stored in DMSO at 10 mg/ml. The cell lines used in this study include HeLa cells (ECACC; Sigma-Aldrich, 93031013), NRK-52E cells (ATCC CRL-1571) and NRK cells stably expressing GFP-tagged GBF1 (described in Zhao et al., 2006). Cells were maintained in Dulbecco's modified Eagle's medium (DMEM) supplemented with 10% FBS, 100 μg/ml penicillin and 100 μg/ml streptomycin in a 5% CO_2_ incubator set at 37°C. During live cell imaging experiments, cells were kept in CO_2_-independent medium supplemented with 10% FBS. Culture medium, CO_2_-independent medium and antibiotics were obtained from GIBCO (Invitrogen, Carlsbad, CA).

The following primary antibodies were used for immunofluorescence experiments: mouse anti-GBF1 (clone 25) (BD Bioscience; 1:1000; lot 31298), sheep anti-TGN46 (AbD Serotec; 1:1000; batch 290914) and mouse anti-P115 (7D1) (Dr Gerry Water; Princeton University, Princeton, NJ; 1:1000). The following primary antibodies were used for immunoblotting experiments: rabbit anti-GBF1 (9D4) (Manolea et al., 2008; 1:500), goat anti-GFP (Eusera; 1:5000, EU3), rabbit anti-GFP (Eusera; 1:50,000; EU1, lot T107), rabbit anti-ManII (Dr Kelley Moremen; University of Georgia, Athens, GA; 1:2000) and mouse anti-tubulin monoclonal (Sigma; 1:1000; lot 096K4777). Secondary antibodies (1:600) for immunofluorescence (Alexa Fluor 546 goat anti-rabbit, lot 1504518; Alexa Fluor 555 donkey anti-sheep, lot 1458623; Alexa Fluor 647 donkey anti-mouse, lot 702339) were used at 1:600 and obtained from Molecular Probes (Invitrogen). Secondary antibodies for immunoblots (Alexa Fluor 680 goat anti-rabbit, lot 1655809; Alexa Fluor 750 goat anti-mouse, lot 1712786; Alexa Fluor 790 donkey anti-goat, lot 41443A) were used at 1:10,000 and were obtained from Molecular Probes.

### Generation of a pEGFP-*Sbf*I-GBF1 truncation library and HeLa cell lines

We generated a library of GBF1 N-terminal and C-terminal truncation constructs based on the published domain and inter-domain boundaries described by Cherfils and colleagues (Mouratou et al., 2005). A schematic diagram of each truncation construct is shown in Fig. 6. The GBF1 truncations were constructed by PCR amplification of the regions of interest from a GBF1 template (Claude et al., 1999) with specific primers that introduced a *Sac*II restriction site at the 5′-end and an *Sbf*I restriction site at the 3′-end. These sites allowed for the directional insertion of GFB1 truncations into a modified pEGFP-C1 vector, to which an *Sbf*I site was added within the *Sma*I site. The *Sbf*I site was introduced by insertion of a synthetic duplex (5′-ATACCTGCAGGTAT-3′; Integrated DNA Technologies, San Diego, CA) in phosphatase-treated (calf intestinal alkaline phosphatase; Invitrogen) *Sma*I cut pEGFP-C1. The resulting plasmids encoded GBF1 truncations bearing an N-terminal EGFP tag containing a 19-residue linker (SGLRSRAQASNSAVDGTAV) contributed by the multiple cloning site. All constructs were sequenced, and the size of encoded chimeras was confirmed to be correct by immunoblotting.

The isolation of inducible HeLa cell lines stably expressing GFP-tagged forms of full-length and truncated GBF1 took advantage of the Flip-In T-Rex system (Invitrogen). First, plasmid pcDNA5/FRT/TO was modified at the *EcoR*V and *Kpn*1/*BamH*1 sites to receive *Sbf*I and *Nhe*1 restriction sites, respectively. The resulting plasmid was then used for insertion of *Nhe*1-*Sbf*I fragments encoding either EGFP-tagged full-length or N885-1586 GBF1. The resulting plasmids were co-transfected with pOG44 into HeLa TREX Flp-In cells obtained from Dr Stephen Taylor (University of Manchester, UK; Tighe et al., 2008). Stable populations were selected and characterized as per the manufacturer's instructions.

### Additional plasmids and recombinant proteins

A plasmid encoding GFP-tagged ERGIC-53 (Ben-Tekaya et al., 2005) was obtained from Dr Hans-Peter Hauri via Dr Hesso Farhan (Department of Biology, University of Konstanz, Switzerland). A plasmid encoding mCherry-ERGIC-53 was constructed by exact substitution of the GFP for mCherry-coding sequences. Plasmids encoding WT and catalytically dead RQ mutations of pEGFP-tagged forms of ArfGAP1, ArfGAP2 and ArfGAP3 were generated as described (Parnis et al., 2006) and obtained from Dr Dan Cassel (Department of Biology, Technion-Israel Institute of Technology, Haifa, Israel). A plasmid encoding an HA-tagged form of Arf1-6-1 (Honda et al., 2005) was a generous gift from Dr Julie Donaldson (NIH, Bethesda, MA). The Arf1-6-1-coding region was substituted into pEGFP-Arf1 (Chun et al., 2008) using standard recombinant DNA technology. A T31N version of this chimera was generated using a modified QuickChange kit as described before (Quilty et al., 2014). ArfGAP1 used in *in vitro* GBF1 recruitment assays was purified as described (Pevzner et al., 2012) and a generous gift from Dr Dan Cassel.

### Cell transfection and imaging experiments

Imaging experiments were performed with tissue culture cells grown on glass coverslips (no. 1.5; Fisher Scientific, Ottawa, ON) in 6-well plates. Coverslips were sterilized by dipping in 70% ethanol and open-flame ignition. Transfection of plasmids (2 µg) for transient protein expression was performed on cells grown to ∼60–80% confluence by using TransIT-LTI transfection reagent (Mirus, Madison, WI) or Lipofectamine 2000 (Invitrogen) according to the manufacturer's instructions; cells were cultured for ∼18 h to allow for protein expression. Immunoblot analysis of cell monolayers transfected with plasmids encoding ArfGAP-GFP was carried out using GFP antibodies and confirmed that similar levels of expression were achieved.

Typically, following treatments, cells were washed in PBS warmed to 37°C and fixed with 3% paraformaldehyde (with 100 μM CaCl_2_ and 100 μM MgCl_2_ in PBS) at 37°C for 20 min. Fixation was halted by incubation in quench buffer (50 mM NH_4_Cl in PBS) for 10 min at room temperature. Subsequently, cells were incubated in permeabilization buffer (0.1% Triton X-100 in PBS) to allow antibody access to intracellular structures. Prior to antibody incubations, cells were blocked in a 0.2% gelatin solution made in PBS. Cells were double-labeled with antibodies of differing species and processed as described previously (Zhao et al., 2002). Note that preliminary analysis revealed inconsistent loss in Golgi localization of GBF1 truncations in cells treated with a variety of fixatives ranging from alcohols to high concentrations of paraformaldehyde. In this case, we examined localization in live cells as described below.

### Fluorescence microscopy

Cells for live-cell microscopy experiments were grown on 25 mm round glass coverslips (no. 1.5; Fisher Scientific) in 6-well dishes. When ready for imaging, coverslips were transferred to Attofluor cell chambers (Invitrogen), and the medium was changed to CO_2_-independent DMEM (Gibco Laboratories, Grand Island, NY) supplemented with 10% FBS (Gemini Bio-Products, Sacramento, CA) before imaging commenced. Where stated, BFA was added to a final concentration of 10 µg/ml.

Fixed-cell samples (Figs 1 and 2) were prepared as described above and imaged using a Zeiss Axiovert 200 M confocal microscope equipped with an UltraVIEW ERS 3E spinning disk confocal head (PerkinElmer, Waltham, MA) and a 63× objective lens (plan-Apocromat, NA=1.4). Images were captured with a 9100-50 electron multiplier CCD digital camera (Hamamatsu, Hamamatsu City, Japan) and processed with Volocity software (PerkinElmer). Where indicated in legends (Fig. 6; Figs S5, S6 and S7), wide-field fluorescence microscopy was performed on live cells using a DeltaVision Elite (GE Healthcare, Buckinghamshire, UK) microscope equipped with a front-illuminated sCMOS camera driven by softWoRx 6 (GE Healthcare) at 37°C (Applied Precision, Mississauga, ONT) using a 60×1.4 NA oil objective (Olympus, Richmond Hill, CAN). A minimum of four fields of view were acquired in rapid succession by programming the automated stage. Experiments involving drug addition were performed by adding 250 μl of medium containing six times the desired drug concentration to the cell chamber containing 1250 μl of medium. Focus was maintained by use of the UltimateFocus feature. Before analysis, images were deconvolved in softWoRx 6 and processed in Fiji (National Institutes of Health, Bethesda, MD). Only cells with intact Golgi expressing the tagged transgene at low levels were selected for imaging.

### Cell fractionation and preparation of cytosol and membranes

NRK cells stably expressing WT or GFP-GBF1 were used for the production of cytosol. Cells were grown on 15 cm tissue culture dishes to confluence and harvested by using trypsin, which was inactivated by two volumes of complete DMEM supplemented with 10% FBS; then cells were pelleted, the supernatant was aspirated and the weight of the cell pellet was measured. Cells were then washed in PBS to remove residual medium components and re-pelleted. Washed cells were then resuspended in four volumes of ice-cold homogenization buffer and placed on ice. Cells were subsequently homogenized by passing 20× through a cell homogenizer (Isobiotech, Heidelberg, Germany) with a 12 μm clearance. The homogenate was then centrifuged at 4°C and 400***g*** for 5 min to pellet nuclei and unbroken cells. The resulting supernatant was then centrifuged at 4°C and 55,000 rpm for 15 min to pellet the microsomes (Thick wall polycarbonate, TLA-120.1 rotor, Optima TLX Benchtop Ultra; Beckman Coulter, Brea, CA). The resulting supernatant was collected, immediately aliquoted and stored at −80°C. Highly stacked Golgi-enriched membranes isolated from the nuclear fraction (WNG) were obtained from rat livers as described (Dominguez et al., 1999).

HeLa cells stably expressing GFP-GBF1 were also used for the production of cytosol. Cells were induced with 2 µg/ml doxycycline for 24 h prior to collection. HeLa cells were processed as described for NRK cells, with the exception that 14 µm clearance was used for homogenization. The resulting cytosols were analyzed for GBF1 content by immunoblotting.

### Immunoblotting

Following protein separation by SDS-PAGE, proteins were transferred to nitrocellulose membranes (GE Healthcare) at 376 mA for 2 h in transfer buffer (25 mM Tris-HCl, 190 mM glycine, 20% v/v methanol, 2.5% v/v isopropanol). Resulting membranes were then blocked in Licor Odyssey Blocking Reagent (LOBR; Licor Biotechnology, Lincoln, NE) for at least 1 h. Blocked membranes were then incubated with primary antibodies in 50% LOBR. Following three washes in PBS, membranes were incubated in fluorescent secondary antibody for 1 h in 25% LOBR, followed by two 10 min TBST (50 mM NaCl, 0.5% v/v Tween-20, 20 mM Tris-HCl, pH 7.5) washes and three 10 min washes with PBS. Membranes were then scanned on a Licor Odyssey scanner (Licor Biotechnology).

Quantification of immunoblots was performed using Licor Odyssey software version 3.1 (Licor Biotechnology). Band intensities were quantified by manually drawing a rectangle around the region of interest, which was automatically corrected for background noise based on the dimmest pixels in a three-pixel region along the edges of the drawn rectangle. The quantified intensities were exported to Excel worksheets (Microsoft, Redmond, WA), where the mean and standard deviation were calculated and Student’s *t*-tests and normalization calculations were performed. For *in vitro* GFB1 recruitment assays, the GFP-GBF1 band intensity was normalized to the ManII band intensity, a measure of the loading error.

### *In vitro* GBF1 recruitment assay

To perform *in vitro* GBF1 recruitment assays, we mixed 5 μl WNG (Dominguez et al., 1999) and 20 μl GFP-GBF1 NRK cell cytosol in a recruitment assay buffer (50 mM KCl, 2.5 mM MgCl_2_, 0.25 M sucrose, 1 mM DTT, 25 mM Hepes pH 7.4). Incubation conditions were selected on the basis of a previously published transport assay (Braell et al., 1984). It is important to note that the cytosol harvested from the NRK cells also contained a significant amount of Arf together with other cytosolic proteins. The volume of the assay was adjusted to 50 μl with distilled water. Samples were then incubated at 37°C for 5 min to allow for GBF1 recruitment. Following incubation, samples were returned to ice and then centrifuged at 4°C (55,000 rpm for 15 min) in thick wall polycarbonate tubes (TLA-120.1 rotor, Optima TLX Benchtop Ultra). Resulting supernatants were then resuspended in SDS-PAGE loading buffer and boiled. Pellets were washed gently with 50 μl PBS to remove residual amounts of cytosol. Pellets were subsequently resuspended in 50 μl of PBS, mixed with 10 µl SDS-PAGE loading buffer and heated to 95°C. Pellet samples were sonicated in a Bioruptor Pico (Diagenode, Denville, NJ) prior to SDS-PAGE electrophoresis. Resulting fractions were then resuspended in SDS-PAGE sample buffer and assessed by anti-GBF1 and anti-ManII immunoblotting. Three replicate experiments were quantified, and the GFP-GBF1 band intensity was normalized to the ManII band intensity to correct for both the amount of membrane in each assay and loading error.

### Image quantification and analysis

Quantification of fixed cells images was carried out using Imaris 8 software (Bitplane Scientific Software, South Windsor, CT), as previously described (Quilty et al., 2014). Briefly, a minimum number of ten cells from each condition was quantified, and this was repeated for each of the three independent replicates. Three-dimensional surfaces were created around areas of interest in selected cells by using the surfaces feature in Imaris 8. The average pixel intensity (Int) values in the surveyed regions, as well as the volumes (Vol) of these regions, were exported into Excel spreadsheets (Microsoft, Redmond, WA). Average Golgi intensity values were corrected for the average value of the cytosolic intensity, and the calculated values for whole-cell intensity were further corrected for the intensity of the image background.

The equation used for correcting the GBF1 signal at the Golgi was: Fraction of signal at Golgi=Golgi Vol (Golgi Int−cytosol Int)/cell Vol (cell Int−background Int).

Graphs were generated using Excel (Microsoft), with the fraction of GBF1 signal at the Golgi for each condition representing the mean of three independent replicates (*n*=3) and the error bars representing the standard deviation (±s.d.) across the selected cells for each condition. Unpaired two-tailed *t*-tests were performed in Excel (Microsoft). Where stated, two-way ANOVAs followed by Tukey HSD *post hoc* tests were performed using R (version 3.4; https://www.r-project.org/).

Line-scan analysis was performed on extended focus images in which colors were corrected to be true and then imported from Volocity into the Fiji software (National Institutes of Health). Line scans were generated using the RGB Profiler plug-in.

Intensity correlation quotient (ICQ) analysis (Li et al., 2004) of live cell images was performed using the Coloc 2 plug-in in Fiji (Schindelin et al., 2012).

## Supplementary Material

Supplementary information
