# Supplementary material for: The Arf-GDP-regulated recruitment of GBF1 to Golgi membranes requires domains HDS1 and HDS2 and a Golgi-localized protein receptor
Source: J Cell Sci. 2018 Apr 19;132(4):jcs208199. doi: 10.1242/jcs.208199 (PMC6398479; doi:10.1242/jcs.208199)
Supplement: Supplementary information [file joces-132-208199-s1.pdf]

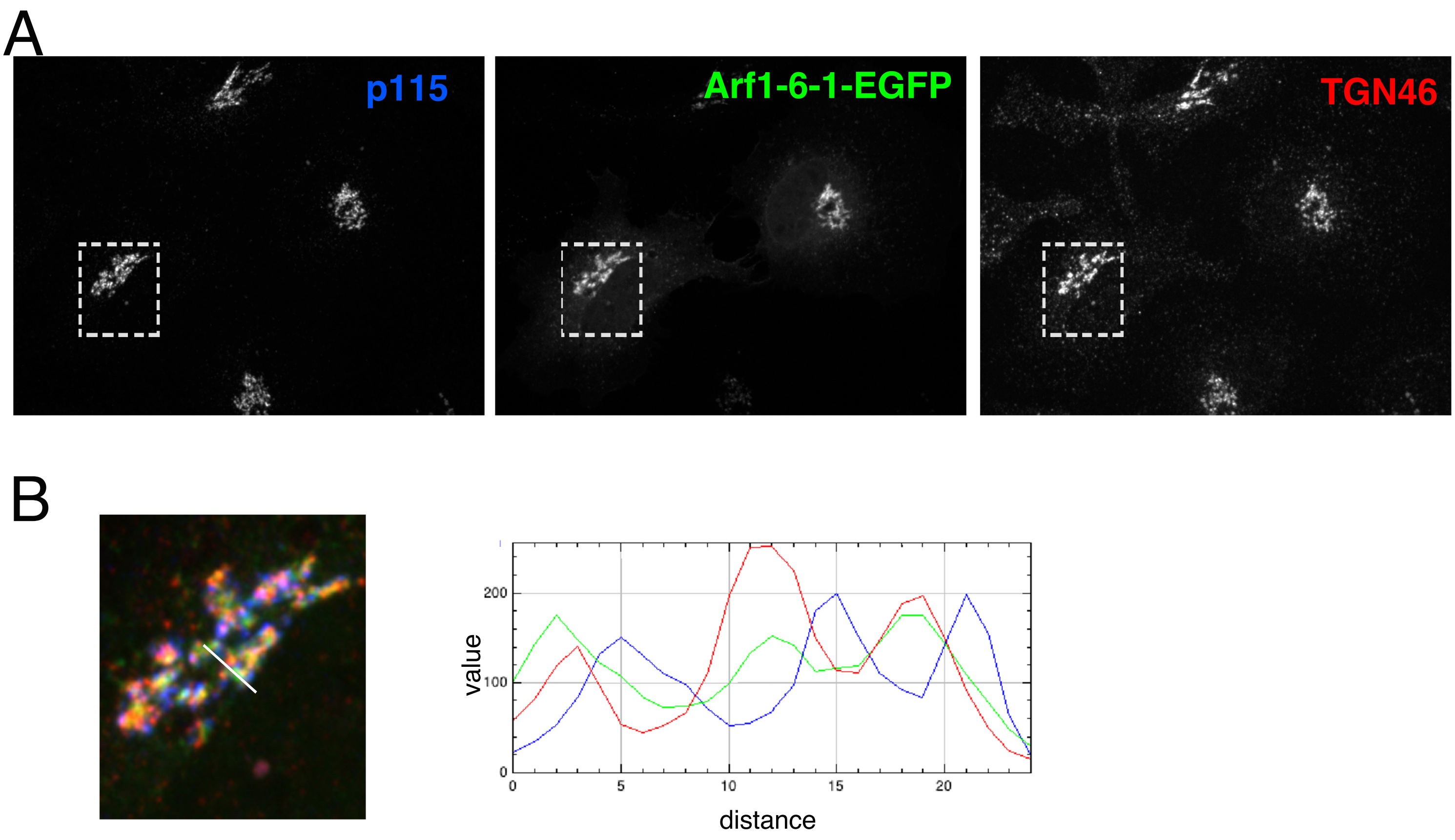

Supplementary Figure S1. Arf1-6-1-EGFP localizes predominantly to TGN membranes. (A) HeLa cells were transfected with WT Arf1-6-1-EGFP (green). Eighteen hours post-transfection, cells were fixed and stained with mouse anti-p115 (blue) and sheep anti-TGN46 (red) antibodies and processed for imaging as described in Methods. The GFP signal best matches the staining pattern observed for TGN46. (B) Scan analysis with RGB_Profiler (FiJi) of magnified representative Golgi images. Position of the scan is indicated by a yellow line. Identical analysis was performed on extended focus images for a minimum of five Golgi images from each of three independent replicates with similar results. This confirms that GFP pattern (green) better matches that with TGN46 (red). Scale bar = 26µm.

Journal of Cell Science • Supplementary information

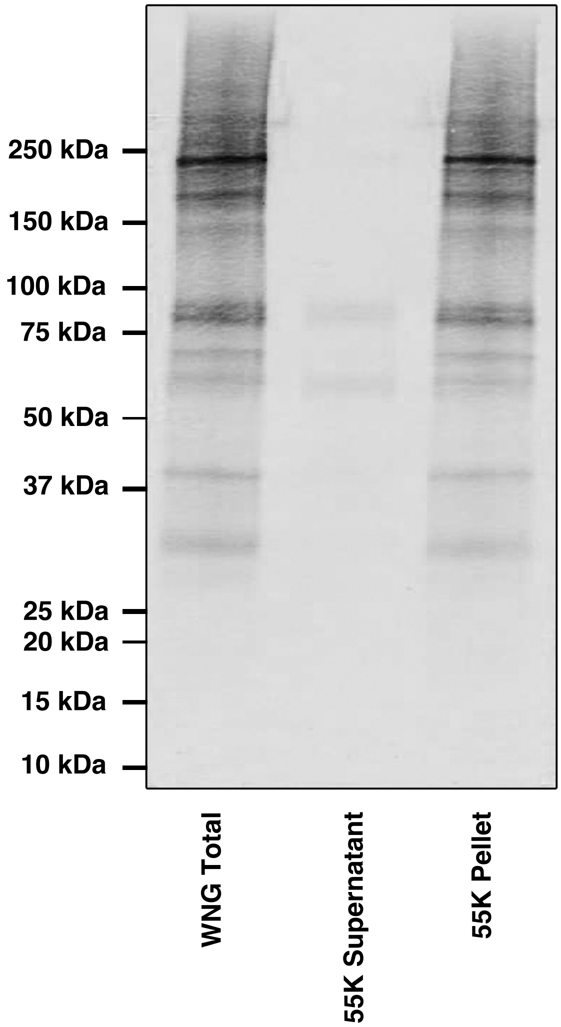

Supplementary Figure S2. GBF1 is present and membrane-associated in WNG preparations. Total WNG membranes along with WNG fractions separated into 55K pellet and supernatant were resolved by SDS-Page. Proteins were transferred to nitrocellulose and immunoblotted with a mouse anti-GBF1 monoclonal antibody and donkey anti-mouse Alexa 750 secondary and was then scanned in a Licor Odyssey scanner. The resulting immunoblot is displayed

Journal of Cell Science • Supplementary information

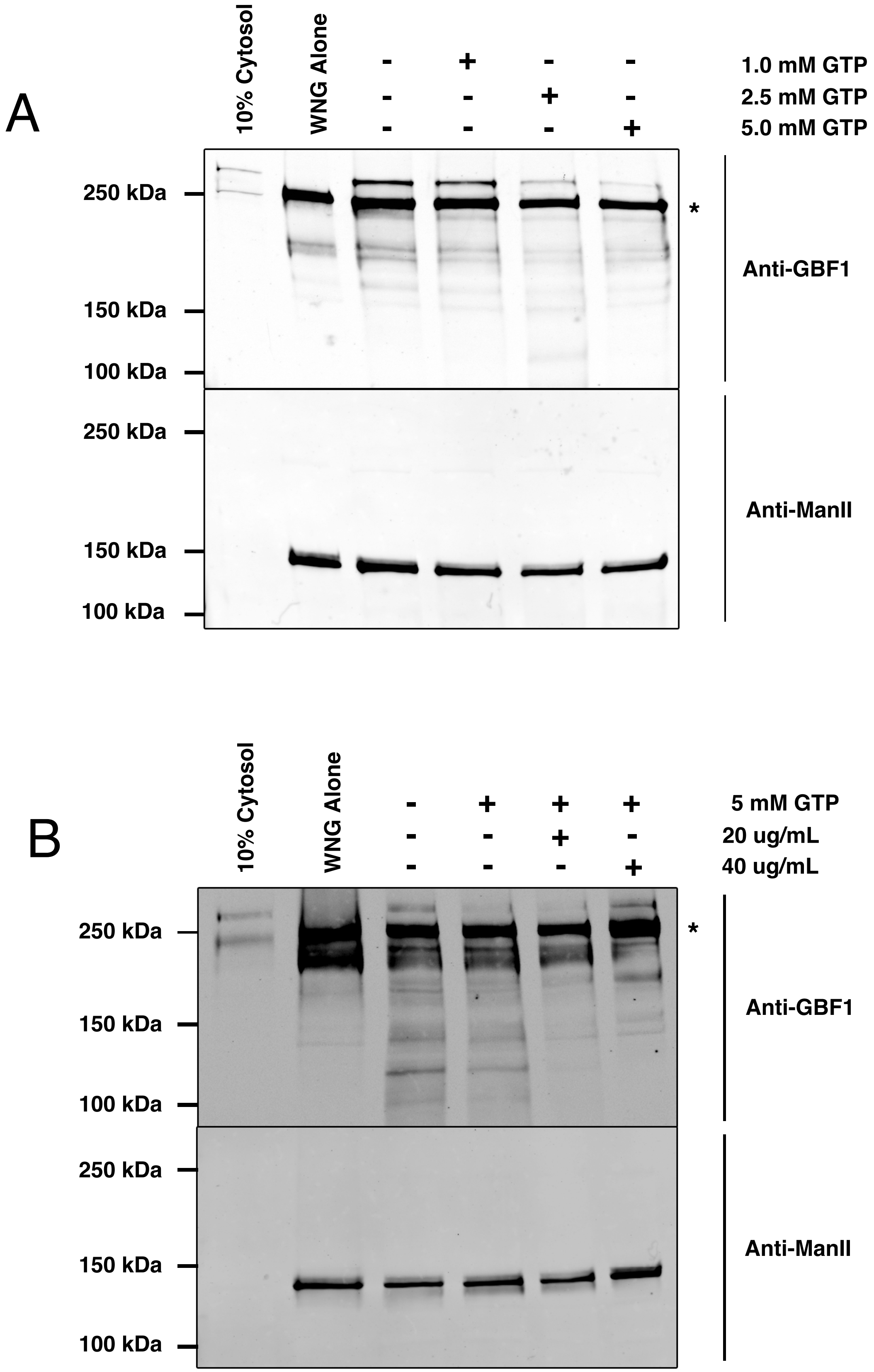

Journal of Cell Science • Supplementary information

Supplementary Figure S3. Immunoblots for GFP-GBF1 Binding Assay with excess GTP or ArfGAP1

WNG membranes were incubated with GFP-GBF1 NRK cytosol with or without the addition of (A) GTP or (B) ArfGAP1, as indicated. Samples were then separated into membrane and supernatant fractions by centrifugation. Resulting pellets were separated by SDS-PAGE along with 10% cytosol and WNG membranes and then analyzed as for Figure 2. A representative scan (Licor Odyssey) of on immunoblot is shown. The * denotes endogenous GBF1.

Journal of Cell Science • Supplementary information

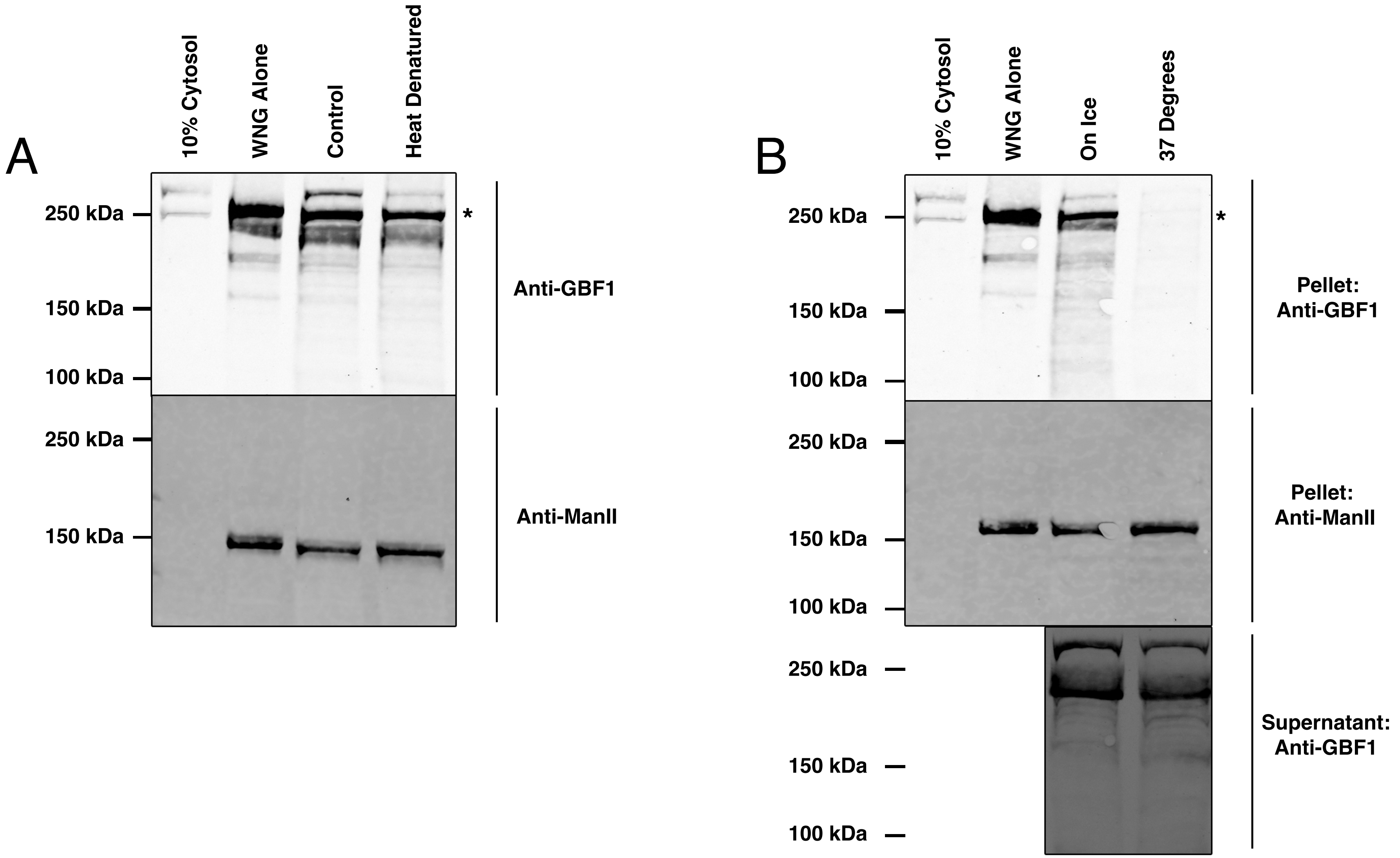
A
10% Cytosol
WNG Alone
Control
Heat Denatured
250 kDa
150 kDa
100 kDa
250 kDa
150 kDa
*
Anti-GBF1
Anti-ManII
B
10% Cytosol
WNG Alone
On Ice
37 Degrees
250 kDa
150 kDa
100 kDa
250 kDa
150 kDa
100 kDa
250 kDa
150 kDa
100 kDa
*
Pellet: Anti-GBF1
Pellet: Anti-ManII
Supernatant: Anti-GBF1

Supplementary Figure S4. **Immunoblots for GFP-GBF1 Binding Assay with heat- or protease-treated membranes**. GFP-GBF1 binding assays were performed as for Figure 2 with the exception that WNG membranes were pre-treated. For panel A, WNG membranes were pre-incubated at 95 degrees Celsius or on ice for 5 minute prior to a binding assay. For panel B, WNG membranes were pre-incubated at 37° Celsius with or without trypsin as described in Methods. Following the binding assay, samples were separated into membrane and supernatant fractions by centrifugation. Resulting pellets and supernatants were separated by SDS-PAGE along with 10% cytosol and WNG membranes and then analyzed as described in Methods with the indicated antibody. A representative scan (Licor Odyssey) of an immunoblot is shown. The * denotes endogenous GBF1.

Journal of Cell Science • Supplementary information

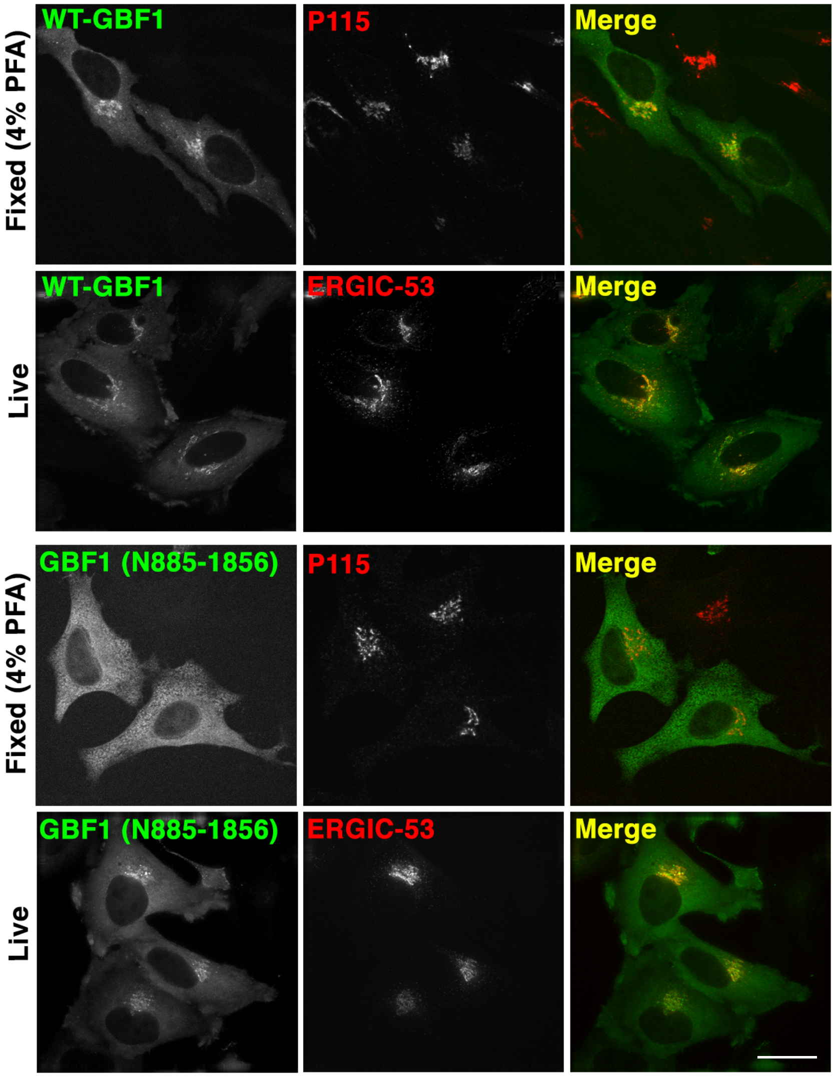

Supplementary Figure S5. GBF1 truncations are most readily imaged in live HeLa cells. HeLa cells were transiently transfected with EGFP-tagged forms of either full length GBF1 or N205-1856-GBF1 and fixed in 4% paraformaldehyde and stained for the cis-Golgi marker p115. Alternatively, HeLa cells were cotransfected with plasmids encoding mCherry-ERGIC53 and either the EGFP-tagged full length or a deletion mutant. Cells were subjected to live cell wide-field fluorescence microscopy and imaged as described in Methods **(A)** Images obtained with cells expressing full length EGFP-GBF1. **(B)** Images obtained with cells expressing EGFP-tagged GBF1 truncation N205-1856. An extended focus view of all acquired z-slices is shown. Scale bar = 26 µm.

Journal of Cell Science • Supplementary information

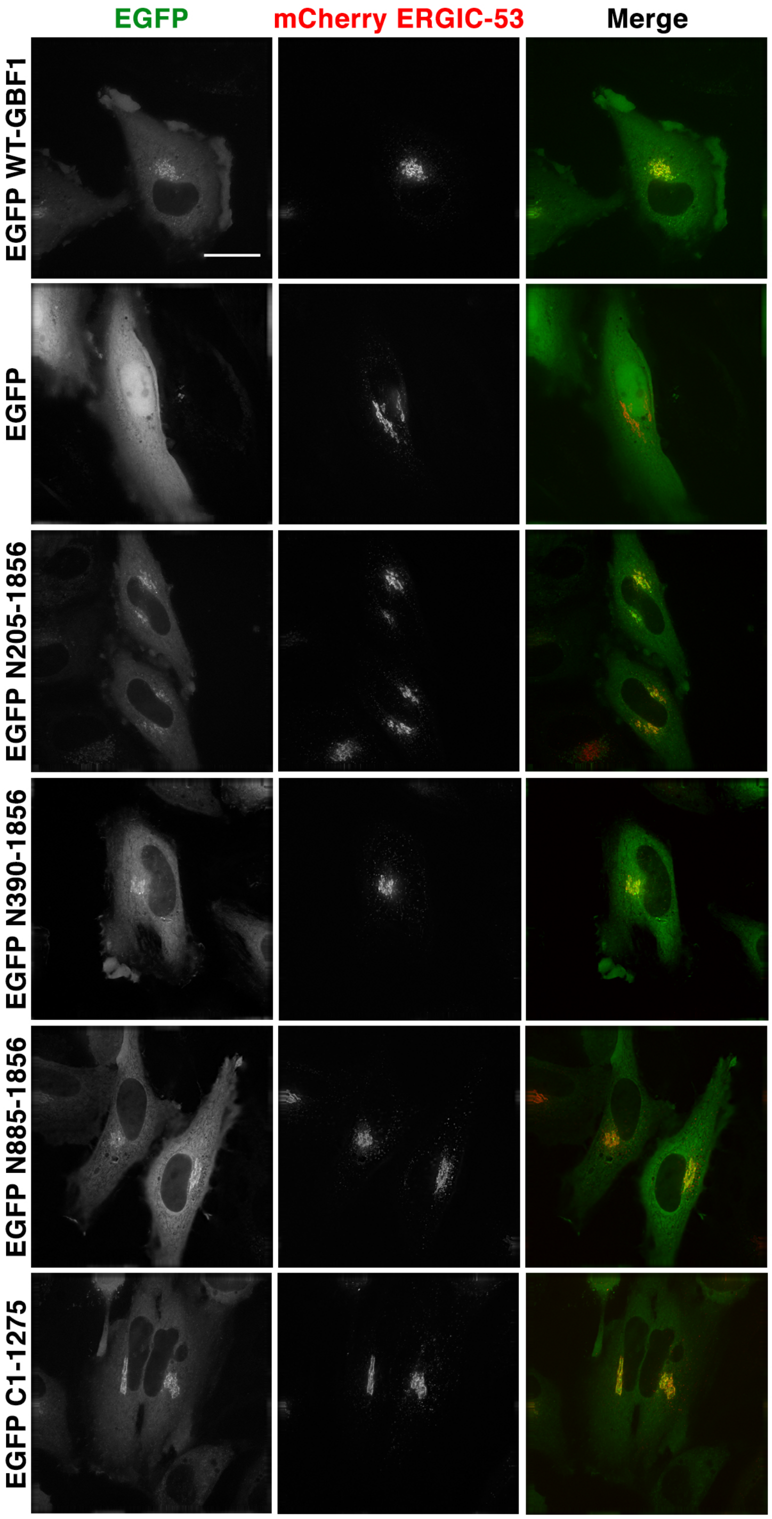

Journal of Cell Science • Supplementary information

Supplementary Figure S6. Distribution of GBF1 truncations. HeLa cells were transfected with plasmids encoding mCherry-ERGIC53 and each of the GBF1 truncations pictured in Figure 6 and then imaged by live cell wide-field fluorescence microscopy. Several of the truncations reproducibly bound to structures positive for mCherry-ERGIC53. Representative images from those 4 truncations obtained in the same replicate are displayed as an extended and deconvolved focus view of z-slices. Images displayed are representative of a minimum of 10 cells from each of three replicate experiments and used for the summary shown in Figure 6. Scale bar = 26 μm.

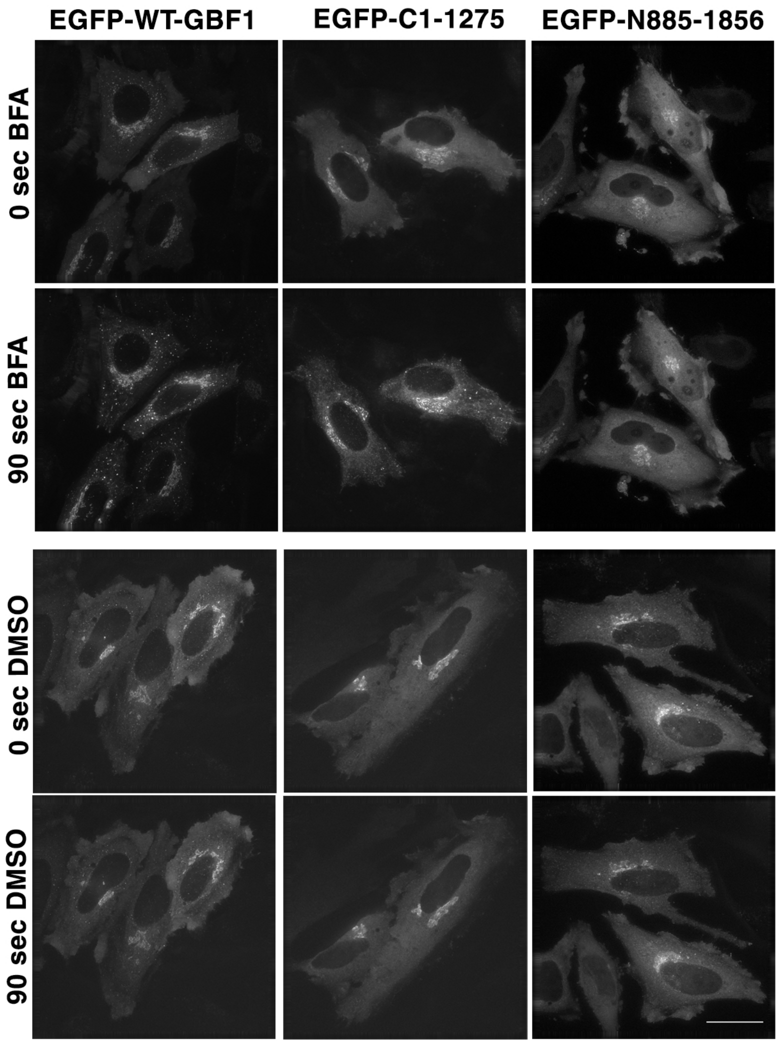

Supplementary Figure S7. Distribution of cytosolic GBF1 truncations in absence and presence of BFA. HeLa cells were transfected with plasmids encoding mCherry-ERGIC53 and GBF1 truncations pictured in Figure 6 and then imaged by live cell wide-field fluorescence microscopy. Representative images from cells expressing the two shortest truncations obtained in the same experiment are displayed as an extended and deconvolved focus view of z-slices. Images displayed are representative of a minimum of 10 cells from each of three replicate experiments. Quantitative analysis of these and similar images is presented in Figure 6B. Scale bar = 26 µm. (**top rows**) BFA was added to a concentration of 10 µg/ml one minute after the start of imaging; (**bottom rows**). Carrier DMSO was added one minute after the start of imaging

Journal of Cell Science • Supplementary information
